# Lack of cardioprotection from subcutaneously and preischemic administered Liraglutide in a closed chest porcine ischemia reperfusion model

**DOI:** 10.1186/1471-2261-9-31

**Published:** 2009-07-23

**Authors:** Jens Kristensen, Ulrik M Mortensen, Morten Schmidt, Peter Haubjerg Nielsen, Torsten Toftegaard Nielsen, Michael Maeng

**Affiliations:** 1 Department of Cardiology, Aarhus University Hospital, Skejby, Brendstrupgaardsvej, DK-8200 Aarhus N, Denmark

## Abstract

**Background:**

Glucagon-like peptide 1 (GLP1) analogues are promising new treatment options for patients with type 2 diabetes, but may have both potentially beneficial and harmful cardiovascular effects. This may also be the case for the analogues of GLP1 for clinical use. The present study examined the effect of treatment with Liraglutide, a long-acting GLP1 analogue, on myocardial ischemia and reperfusion in a porcine model.

**Methods:**

Danish Landrace Pigs (70–80 kg) were randomly assigned to Liraglutide (10 μg/kg) or control treatment given daily for three days before ischemia-reperfusion. Ischemia was induced by balloon occlusion of the left anterior descending artery for 40 minutes followed by 2.5 hours of reperfusion. The primary outcome parameter was infarct size in relation to the ischemic region at risk. Secondary endpoints were the hemodynamic parameters mean pulmonary pressure, cardiac output, pulmonary capillary wedge pressure as measured by a Swan-Ganz catheter as well as arterial pressure and heart rate.

**Results:**

The infarct size in relation to ischemic risk region in the control versus the Liraglutide group did not differ significantly: 0.46 ± 0.14 and 0.54 ± 0.12) (mean and standard deviation (SD), p = 0.21). Heart rate was significantly higher in the Liraglutide group during the experiment, while the other hemodynamic parameters did not differ significantly.

**Conclusion:**

Liraglutide has a neutral effect on myocardial infarct size in a porcine ischemia-reperfusion model.

## Background

Liraglutide, a long-acting Glucagon-like peptide 1 (GLP1) analogue, allowing once daily administration in contrast to genuine GLP1, is a promising new treatment option for patients with type 2 diabetes [1]. This incretin hormone stimulates beta cell insulin secretion in a glucose-dependent manner, lowers glucagon, reduces appetite and reduces body weight, effects beneficial in type 2 diabetic patients [2]. Since the risk of coronary heart disease is increased 2 to 4 times in diabetic patients [3], it is of importance to assess if ongoing Liraglutide treatment has any deleterious or beneficial effect on myocardium exposed to ischemia. Especially because effects of GLP1 has been reported in other organs than the pancreatic islets, e.g. the heart [4].

Reports exist evaluating cardiac effects of GLP1 in rodents [5-7] as well as a study in a porcine model giving GLP1 as short-term infusions [8]. Some of these studies report beneficial effects in myocardial ischemia-reperfusion injury using genuine GLP1. A recent study reports similar effects with a GLP1 analogue in an isolated rat heart model [9]. Furthermore a recent study in mice reports benefical effects by Liraglutide administration for 7 days before myocardial ischemia [10]. However, few data on the analogue Liraglutide in larger animals exists and with clinical relevant administration of Liraglutide by subcutaneous injections. A recent study with another GLP1 analog Exanatide has reported beneficial effects [11]. In this study exanatide was given after 75 min of coronary ligation. Results may differ in relation to timing of treatment intervention.

GLP1 has been reported to increase heart rate and blood pressure [12] in experimental models, possibly by increasing intracellular cAMP with receptor stimulation. Such effects could be harmful in myocardial ischemia. The effects may also differ slightly between GLP1 and analogues such as Liraglutide. Since both potential harmful effects such as heart rate and blood pressure increase and beneficial effects in relation to cardiovascular effects have been reported further studies are needed.

In the present study we assessed the effect of occlusive myocardial ischemia and subsequent reperfusion in pigs given subcutaneous Liraglutide daily for three days before the ischemic insult.

## Methods

The protocol for the animal experiments was performed according to the guidelines in the Guide for the Care and Use of Laboratory Animals and was approved by the National Committee on Animal Research Ethics (Copenhagen, Denmark).

Twenty-four 6-months old pigs of mixed Danish Landrace and Yorkshire breeds with a body weight of 70–80 kg were used. Four animals did not enter the analysis: One animal suffered ventricular fibrillation during the initial catheterisation (control animal) before index ischemia and was excluded for this reason, and another animal was excluded due to rupture of the PTCA balloon during the index ischemia (Liraglutide group). One animal died during the initial anaesthesia (Liraglutide group), without obvious reason. Another animal was excluded due to myocardial ischemia before the index ischemia as detected on the vector electrographic tracing and was presumably ischemic preconditioned before the index ischemia, as judged by vector electrocardiography and for this reason excluded (control group). The animals were sedated with intramuscular injections of 100 mg of midazolam (1,33 mg/kg) plus 500 mg of ketamine (6,66 mg/kg) and then by intravenous injection of 1000 mg pentobarbital. The anesthesia was maintained by intravenous infusion of 900 mg/hour pentobarbital. After intubation, the animals were ventilated on a respirator (Elema-Siemens, Solna, Sweden) using 8.5 L/min air/oxygen (50%/50%), adjusted according to arterial blood gas analysis repeatedly during the experiment (ABL Radiometer A/S, Denmark). Potassium-glucose (20 mmol potassium/500 ml glucose 5%) infusion at 50 ml/h, was infused throughout the experiment. With this infusion rate, blood glucose was kept in physiological ranges equally in both groups. Rectal temperature and electrolytes were maintained in physiological ranges in the two groups during the experiment. All animals received 500 ml/h isotonic NaCl infusion throughout the entire study to maintain normovolaemia (to meet the estimated fluid requirements for pigs of the given size during ventilator treatment).

### Dosing of Liraglutide/vehicle

Liraglutide (supplied by Novo-Nordisk A/S, Denmark) was given from 3 days before the occlusion of the coronary artery. 10 microg/kg was injected subcutaneously once daily based on suggested dose for patients with type 2 DM and resulted in comparable concentration intervals to a previous study in humans [13]. Plasma samples (in EDTA) for Liraglutide measurement were taken just before coronary occlusion and measured by Novo-Nordisk, Denmark using a two side immunoassay method [13].

### Experimental protocol

The experiment was performed in a randomised manner with the investigators blinded to the treatment. The treatment code was revealed only just before the statistical analysis.

The right carotid artery and the neck veins were surgically exposed and 6F introducer sheaths inserted for arterial and venous access. The rectal temperature was stabilised between 38°C and 38.5°C by heating with blankets or cooling with ice packs. After stabilisation, baseline values (temperature, heart rate, arterial blood pressure and cardiac output) were registered. Occlusive ischemia was induced after the stabilisation period for 40 minutes by inflation of a conventional angioplasty balloon in the left anterior descending coronary artery (LAD) after the 2^nd ^diagonal branch. The correct position of the balloon was controlled by angiography immediately after balloon inflation and just prior to balloon deflation. After balloon deflation, a final angiogram was performed to ensure restoration of coronary flow. After 2.5 h of reperfusion, thoracothomy was performed to excise the heart after demarcation of the ischemic region at risk.

### Vector-electrocardiography

Automatic computerized Vector-electrocardiography (Midas, Ortivus, Täby, Sweden) was used to monitor the magnitude of ST vector deviations during the entire procedure [14]. We thereby had an effective control of persistent occlusion during ischemia and reperfusion and procedure-related unwanted ischemic complications would therefore be readily detected. During the run-in period, the baseline ST vector magnitude was measured during the 5 minutes prior to the angioplasty balloon deployment.

### Arrhythmia Surveillance

Monitoring was performed by a 2-channel tape recorder (Tracker, Reynolds, Hertford, UK). We analysed the tapes using the Pathfinder 700 electrocardiography analysis system (Reynolds). The numbers of episodes of ventricular fibrillation and the time from start of ischemia to first episode of ventricular fibrillation were registered.

### Hemodynamic and basic parameters

Rectal temperature was monitored continuously by a rectal probe. Using the jugular vein introducer sheath, a Swan-Ganz catheter with continuous cardiac output (CO) monitoring (by continuous thermodilution by a thermofilament on the standard clinical-use Swan-Ganz catheter) was placed in a pulmonary artery and used to measure cardiac output (using a Edwards Vigilance monitor, Horw, Switzerland), central venous pressure (CVP), mean pulmonary artery pressure (PAP), and pulmonary capillary wedge pressure (PCWP). Heart rate (HR) was measured and blood pressure (MABP) was obtained by arterial catheterisation. All parameters were measured at baseline, at 15 and 35 minutes during ischemia, and at 60 and 120 minutes of reperfusion.

### Blood gas and blood glucose measurements

Arterial blood analyses (includes blood glucose) was made immediately after sheath placement (= before glucose/potassium infusion), at baseline, at the end of ischemia, and at 60 min and 120 min of reperfusion (ABL Radiometer A/S, Denmark).

### Assessment of Infarct Size

Measurement of infarct size (IS) and area at risk (AAR) was performed as described below. A midline sternotomy was performed after 2.5 hours of reperfusion, followed by ligation of the LAD just distal to the second diagonal branch. Fluoresceine (Acros Organics, Geel, Belgium) was injected into the left auricle for in vivo demarcation of the myocardial AAR. After excision of the heart, the left ventricle (LV) was weighed and sliced in 8 mm thick slices perpendicular to the LAD, placed under a Wood's lamp, and photographed with a digital camera (Camedia 4040, Olympus Optical Co., Tokyo, Japan). After incubation in 800 ml 2,3,5-triphenytetrazolium chloride (ICN biomedicals Inc., Aurora, Ohio, 1% W/V phosphate buffer solution) for 10–15 min at 37°C for demarcation of IS, the slices were re-photographed. Using digital planimetry (analysis 3.0, Münster, Germany) the proportions of AAR and IS to the cut surface of each section was determined. The mass of AAR and IS was calculated by multiplying the mass of each section with the respective proportion.

### Statistics

A to sided t-test was applied for the two group comparison of histochemical data. The hemodynamic parameters measured were analysed using univariate ANOVA for repeated measurements (STATA statistical software package) with emphasis on differences in change over time with intervention between groups. Data are presented as mean +/- standard deviation (SD). A value of p < 0.05 was considered statistically significant. Normal distribution of the data was tested by the Kolmogorov-Smirnov Normality test.

## Results

### Myocardial infarct size

**Figure 1:**
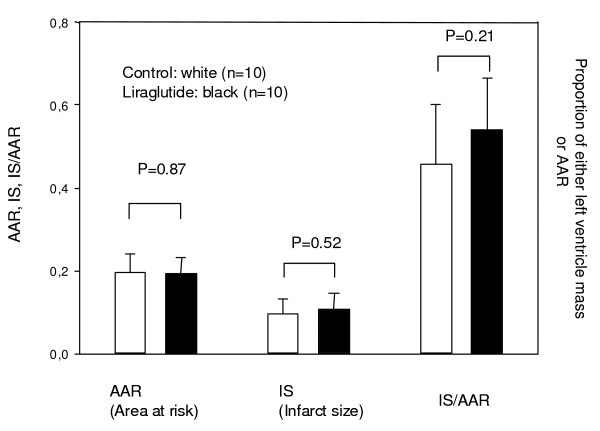
**Ischemic region at risk (AAR), Infarct size (IS) and the infarct size in relation to the region at risk (IS/AAR) are shown as measured by histochemistry**. The unit on the y-axis is the proportion of either left ventricular mass (AAR, IS) or the IS as a proportion of the AAR. P values follow from the figure. Values are mean +/- SD.

Data on infarct size (IS), area at risk (AAR) and the infarct size in relation to the area at risk (IS/AAR) are shown in figure 1. There was no significant differences in neither AAR, IS nor IS/AAR.

### Hemodynamic data

**Figure 2:**
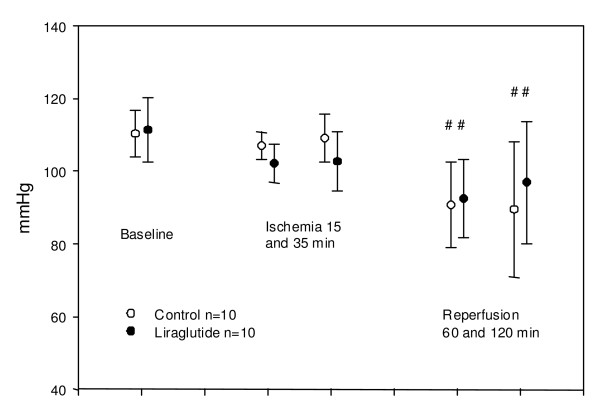
**Mean blood pressures at indicated time**. No significant difference in change over time between groups. No significant difference in level between groups (p = 0.07 and 0.21 resp.) #: Both control and liraglutide at 60 and 120 min significantly lower than compared to respective baseline values (p < 0.05). Values are mean +/- SD.

**Figure 3:**
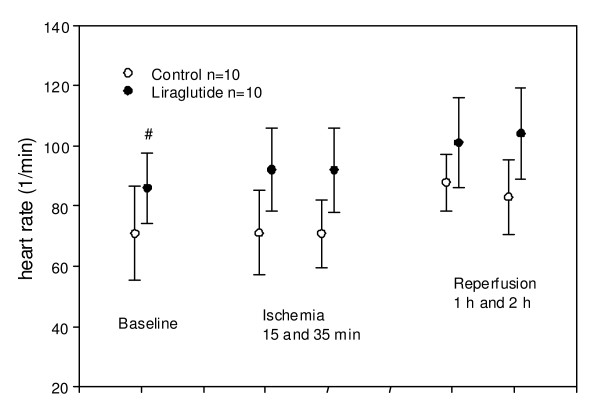
**Heart rate at indicated time**. #: The heart rate level is significantly larger in liraglutide group than control group throughout (p = 0.03) the experiment, while there is no significant difference in the change over time between groups (p = 0.21). Values are mean +/- SD.

**Figure 4:**
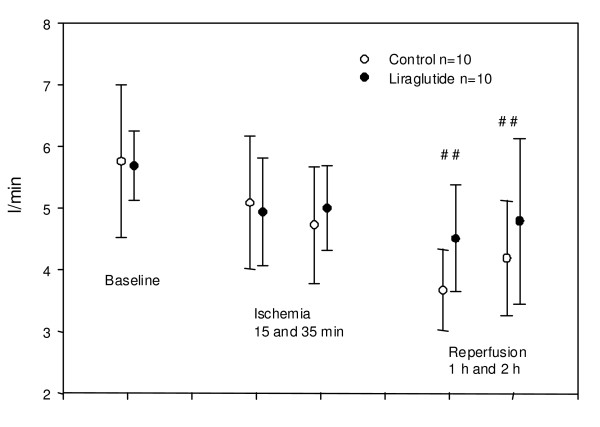
**Cardiac output at indicated time**. No significant difference between control and liraglutide group. #: significant decline in values at reperfusion 60 and 120 min vs. baseline in both control and intervention groups (p < 0.05), but no difference between groups. Values are mean +/- SD.

**Figure 5:**
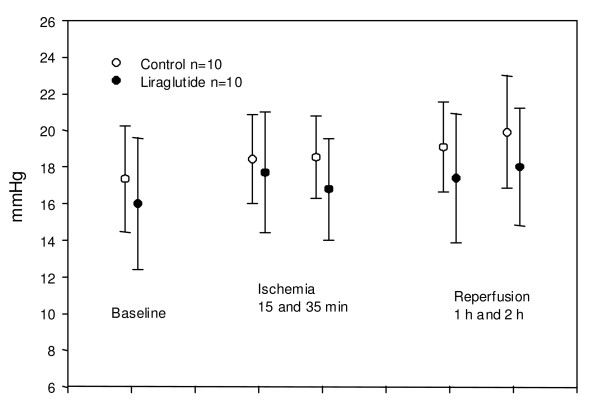
**Mean pulmonary pressure**. No significant difference in change over time between group, and no significant difference in level between groups (p = 0.49 and 0.59). Values are mean +/- SD.

Results of measurements of mean arterial blood pressure (MABP), heart rate, cardiac output and mean pulmonary pressure are shown in figures 2, 3, 4 and 5. Liraglutide did not affect MABP (figure 2).

Apart from changes in hemodynamic parameters attributable to the ischemic insult per se (such as similar decreases in cardiac output in the two groups during the experiment), the only parameter differing significantly between groups was heart rate. The level of this parameter was generally higher in the Liraglutide group during the entire experiment (figure 3). There was no significant difference in the evolution of this parameter during the experiment between groups. Central venous pressure, pulmonary capillary wedge pressure and the derived parameters pulmonary vascular resistance and systemic vascular resistance did not differ between groups (data not shown). Neither the proportion of animals suffering ventricular fibrillation nor the time to VF was significantly different between groups. During the index ischemia 3 animals in both the control group and in the Liraglutide group suffered ventricular fibrillation and were successfully defibrillated with 1 or 2 DC shocks (360 J). No animals had ventricular fibrillation during reperfusion.

Liraglutide concentrations were below detection level in control group and 6738 pM (SEM: 1224 pM) in the treated animals measured just before coronary occlusion.

## Discussion

As patients with diabetes have a significantly increased risk of coronary heart disease including risk of myocardial infarction [3], it is of major importance that new treatment options do not increase myocardial infarct size during ischemia or increase the risk of arrhythmia during ischemia. Liraglutide, a long-acting GLP1 analogue, is a promising new treatment option for patients with type 2 diabetes. There is, however currently limited data on the cardiovascular effects of especially the GLP1 analogues.

In the present study, we examined the effect of Liraglutide administered subcutaneously as clinically intended, in contrast to previous experimental studies with intravenous infusions immediately before, during or after ischemia. The effect was neutral on myocardial infarct size in our porcine model. Heart rate was the only parameter differing significantly between the groups. This is in accordance with a previous experimental study reporting effects of GLP1 on heart rate [12]. Blood pressure did not differ significantly.

The obtained plasma concentrations of Liraglutide immediately before ischemia was comparable to the same body weight adjusted doses in humans [15].

Previously it has been reported in patients given GLP1 infusion in the reperfusion phase after successful PCI based reperfusion, that ejection fraction (EF) increased in the treated group [16]. Whether this effect was due to inotropic effects previously reported for GLP1 or a true reduction of reperfusion injury is not obvious, and furthermore, the mentioned study must be interpreted cautiously because it was a non-randomised study with 10 and 11 patients in the groups. However the effect of GLP1 on myocardial ischemia may differ depending on whether the ischemic insult is occurring during ongoing treatment with GLP1 (or its analogues) or whether the treatment is instituted after the ischemic insult.

A previous porcine study with GLP1 infusion during and just prior to ischemia reported equal infarct sizes in treated and control groups [8]. This is in accordance with our study on Liraglutide. A recent study in a porcine model using Exanatide showed beneficial effects when administered at reperfusion [11]. Both timing of treatment as well as dosing regimen may thus influence the effect as well as the specific analogue.

Studies in rodent models have shown reduction in infarct size in animals given GLP1 in both in-vivo models and in Langendorff-perfused rat hearts [5,6]. It is, however, well known that results may differ considerably between animal models. Larger animal models such as pigs are probably more predictive of results in humans [17].

GLP1 has, as mentioned, been reported to possess inotropic and chronotropic effects, as well as preconditioning-like effects [18]. Such effects are in accordance with the reported intracellular increase in cAMP levels in cardiomyocytes, possibly mediating these responses. It is well known that preischemic intermittent exposure to sympathomimetic effects can act as an ischemic preconditioning stimulus [19,20]. However continued activation of the sympathetic system and inotropic and chronotropic effects, leading to increased myocardial oxygen requirements, are certainly not beneficial in myocardial ischemia. The possibility therefore exists that continued exposure to GLP1 and analogues could be potentially harmful in myocardial ischemia. This issue is therefore obviously very important to clarify.

Despite that heart rate was significantly larger in the treated group, the infarct sizes did not differ significantly and liraglutide therefore seems to be safe to use in this respect.

We chose a dosing regimen giving injections on a daily basis on three previous days before the ischemic insult. The cardiovascular effects could very well be different on such a dosage scheme compared to intravenous infusions as in most of the previous studies. However, subcutaneous injections are the intended route of administration with this GLP1 analogue. It cannot be excluded that differences could be anticipated with long-term administration. Additionally, effects may differ between GLP1 and the analogues such as Liraglutide.

Even though we did not include a direct measure of myocardial contractility, the indirect measures such as blood pressure and cardiac output did not differ between groups. The pigs used in the present study are non-diabetic animals. It is possible that diabetic subjects may differ in the response compared to healthy subjects. However, to our knowledge no porcine model of type 2 diabetes exists, and models using streptozocin etc simulate type 1 diabetes, and Liraglutide treatment is not intended for type 1 diabetes. Furthermore, rodent studies reporting beneficial effects in myocardial ischemia-reperfusion injury suggests an ischemic preconditioning-like effect of GLP1. This raises the possibility, that GLP1 analogues could gain use in a broader category of patients than diabetics, if such effects could be substantiated in larger animal models and humans. It is therefore necessary to conduct studies in both non-diabetic and diabetic animals.

The study is an acute study with no long-term follow up. However, since the treatment with Liraglutide was ongoing before the insult and during ischemia, it would be expected that potential effects would be manifested early. We assessed infarct size early, but we have previously found in the present model, that infarct size does not differ significantly when measured after 3 days [21]. Finally, the porcine model, as compared to rodent models, may be less sensitive for demonstrating cardioprotection. We have, however, previously demonstrated myocardioprotective effects in our porcine model [14,22].

## Conclusion

The present study suggests that Liraglutide, a long-acting GLP1 analogue, administered in a clinically relevant fashion, as ongoing therapy during myocardial ischemia does not induce cardioprotection as judged from the present animal model. Possibly Liraglutide may induce a slight increase in heart rate as has been observed for GLP1.

## Competing interests

The study was funded by Novo-Nordisk, Denmark. The protocol and performance of the study was, however, performed independently from Novo-Nordisk. This includes manuscript preparation and potential article processing fee. Besides this, the authors have no financial or non-financial competing interests.

## Authors' contributions

JK: Preparation of manuscript, design of study, data analysis and interpretation, performance of experiments/acquisition of data. UMM: Design of study, data analysis and interpretation, performance of experiments/acquisition of data, revision of manuscript. MS: Performance of experiments/acquisition of data, revision of manuscript. PH: Performance of experiments/acquisition of data, revision of manuscript. TTN: Design of study, data analysis and interpretation, revision of manuscript. MM: Design of study, data analysis and interpretation, performance of experiments/acquisition of data, revision of manuscript. All authors approved the final manuscript version.

## Pre-publication history

The pre-publication history for this paper can be accessed here:


